# Supplementary material for: Bioactivities and Chemotaxonomy of Four Heracleum Species: A Comparative Study Across Plant Parts
Source: Pharmaceuticals (Basel). 2025 Apr 16;18(4):576. doi: 10.3390/ph18040576 (PMC12030304; doi:10.3390/ph18040576)
Supplement: Supplementary file 1 [file pharmaceuticals-18-00576-s001.zip › pharmaceuticals-3460205-supplementary.pdf]

Table S1. Total phenolic and flavonoid content and yield of methanol extracts.

Plant Extracts		Yield of extract (g/100g)	Total phenolic content (mg GAE[a]/g extract±SD[c])	Total flavonoid content (mg QE[b]/g extract±SD[c])
H. crenatifolium	aerial parts	6.41	15.294±0.351	67.395±2.589
	roots	14.65	289.892±7.753	159.085±3.894
H. paphlagonicum	aerial parts	11.38	29.893±0.995	7.021±1.522
	roots	12.6	179.453±2.408	155.844±4.196
H. sphondylium subsp. *montanum*	aerial parts	7.05	6.195±0.089	68.674±0.878
	roots	9.94	4.023±0.902	142.520±1.74
H. pastinacifolium subsp. *incanum*	aerial parts	12.49	37.720±1.383	70.696±1.696
	roots	23.93	335.744±8.183	154.773±4.506

[a]**GAE:** Gallic acid equivalent; [b]**QE:** Quercetin equivalent; [c]**SD:** Standart deviation.

Table S2. Essential oil compounds of *Heracleum* species.

RRI	Compound	A %	B %	C %	D %	E %	F %	G %	H %	K %	L %	M %	N %
970	Isopropyl isobutyrate	-	-	-	0.4	-	-	-	-	-	-	-	-
1032	α-Pinene	-	-	tr	0.2	0.6	1.6	0.2	tr	1.6	0.4	0.3	0.1
1035	α-Thujene	-	-	-	e	-	-	-	-	1.0	tr	-	-
1061	Isopropyl-2-methylbutyrate	-	-	-	0.5	0.1	0.1	0.3	tr	0.3	0.1	-	tr
1076	Camphene	-	-	-	tr	0.1	0.3	-	-	0.4	0.3	0.3	0.1
1083	Isopropyl isovalerate	-	-	-	0.4	tr	0.1	-	-	-	tr	-	-
1093	Hexanal	-	-	-	tr	-	-	-	-	0.4	tr	-	-
1118	β-Pinene	-	-	tr	0.1	0.2	0.9	0.1	-	0.5	0.2	0.2	0.1
1132	Sabinene	-	-	-	0.1	tr	-	-	-	0.4	tr	tr	tr
1159	Butyl isobutyrate	-	-	-	0.2	-	-	-	-	-	-	-	-
1170	Isobutyl butyrate	-	-	-	0.1	-	-	-	-	-	0.1	-	-
1174	Myrcene	-	-	tr	0.1	0.1	0.3	-	-	0.2	0.1	0.2	0.1
1176	α-Phellandrene	-	-	-	-	-	tr	-	-	-	-	-	-
1185	Isobutyl 2-methylbutyrate	-	-	-	0.1	-	-	0.1	-		tr	-	-
1188	α-Terpinene	-	-	-	tr	-	tr		-	0.1	-	-	-
1194	Heptanal	-	-	tr	-	-	-	-	-	0.1	-	-	0.1
1195	Dehydro-1,8-cineole	-	-	-	-	-	tr	-	-	-	-	-	-
1198	Isobutyl isovalerate	-	-	-	tr	-	-	-	-	-	-	-	-
1203	Limonene	-	-	0.1	0.1	0.2	0.3	0.1	-	1.9	0.4	0.6	0.2
1218	β-Phellandrene	-	-	-	-	-	0.1	-	-	-	-	tr	tr
1230	Butyl butyrate	-	-	-	-	-	-	-	-	-	0.1	-	tr
1241	Butyl 2-methylbutyrate	-	-	-	0.2	-	-	tr	-	-	tr	-	-
1244	2-Pentyl furan	-	-	-	-	-	-	-	-	0.2	-	-	-

1246	(*Z*)-*β*-Ocimene	-	-	0.1	-	0.2	0.8	0.1	tr	0.2	0.1	0.4	0.1
1255	*γ*-Terpinene	-	-	-	4.3	1.8	0.5	0.1	tr	0.2	tr	0.1	tr
1266	(*E*)-*β*-Ocimene	-	-	tr	-	0.1	0.5	tr	0.1	-	tr	0.1	tr
1274	2-methylbutyl butyrate	-	-	-	tr	-	-	-	-	-	0.5	0.1	0.1
1280	*p*-Cymene	-	-	tr	1.0	0.9	0.5	tr	-	0.6	-	0.1	-
1282	Hexyl acetate	0.1	-	-	1.0	-	-	-	tr	-	0.2	-	0.1
1288	2-Methylbutyl isovalerate	-	-	-	tr	-	-	-	-	-	-	-	-
1290	Terpinolene		-	-	-	0.1	0.2	0.1	-	1.3	0.1	0.2	-
1296	Octanal	0.4	0.1	tr	0.2	0.1	0.1	0.2	tr	-	0.2	0.1	0.1
1350	Hexyl propionate	-	-	-	0.3	tr	-	-	-	-	-	-	-
1353	Hexyl isobutyrate	tr	-	-	4.1	1.1	0.2	0.1	tr	-	0.7	0.1	0.1
1360	1-Hexanol	tr	-	-	0.1	-	-	-	-	-	0.3	-	0.1
1384	Heptyl acetate	tr	-	-	-	-	-	tr	-	-	-	-	-
1424	Hexyl butyrate	-	-	-	**12.9**	3.1	0.7	1.7	4.1	2.7	**37.0**	4.1	4.5
1438	Hexyl-2-methyl butyrate	0.2	-	-	3.6	0.9	0.2	0.1	-	tr	0.9	-	0.1
1457	Hexyl isovalerate	0.3	0.2	-	0.4	-	-	-	-	-	0.1	-	-
1497	(*Z*)-3-Hexenyl isovalerate	-	-	-	-	-	-	-	-	-	0.1	-	-
1503	2-Methoxy-3-sec-butyl pyrazine	-	-	-	-	-	0.1	-	-	-	-	-	-
1483	Octyl acetate	**95.4**	**85.9**	**9.5**	**39.4**	**11.3**	3.7	**73.7**	**56.1**	**57.1**	**15.6**	6.5	2.6
1506	Decanal	0.1	-	-	0.1	-	-	0.2	tr	tr	0.2	0.1	-
1516	(*Z*)-4-Octenyl acetate	0.1	-	-	3.5	0.8	0.2	0.9	0.7	0.9	-	0.1	-
1525	Heptyl butyrate	-	-	-	-	-	-	-	-	-	tr	-	-
1535	2-Isobutyl-3-metoxy pyrazine	-	-	-	-	-	tr	-	-	-	-	-	-
1535	*β*-Bourbonene	-	-	-	-	-	-	-	-	-	-	0.1	-
1547	Octyl isobutyrate	-	-	-	1.5	0.3	0.1	-	-	-	0.6	-	-

1552	(*Z*)-4-Octenol	-	-	-	0.2	-	-	-	-	-	-	-	-
1553	Linalool	-	-	-	0.5	0.2	-	-	-	-	0.1	0.3	-
1562	Octanol	3.4	2.4	0.3	0.9	0.8	0.3	2.3	6.7	8.2	0.9	0.6	0.4
1571	trans-p-Mentha-2-en-1-ol	-	-	-	-	-	0.1	-	-	-	-	-	-
1583	Longifolene (=Junipene)	-	-	-	-	-	-	-	-	-	0.2	0.2	1.6
1590	Bornyl acetate	-	tr	tr	-	-	-	-	0.4	tr	2.6	7.3	2.3
1592	Longiborneol	-	-	-	-	-	-	-	-	-	-	-	0.3
1614	Carvacrol methyl ether	-	-	-	-	-	-	-	-	-	-	0.4	-
1617	Lavandulyl acetate	-	-	-	-	-	-	-	-	-	0.3	1.0	0.5
1617	Hexyl hexanoate	-	-	-	1.1	tr	-	0.1	-	-	1.1	0.3	-
1623	Octyl butyrate	-	-	-	0.6	tr	-	6.3	3.3	2.6	**20.6**	2.0	1.7
1634	Octyl-2-methyl butyrate	tr	-	-	1.1	tr	-	0.1	-	-	0.5	-	-
1644	Widdrene	-	-	-	-	-	-	-	-	-	0.1	0.4	3.1
1655	Octyl isovalerate	tr	-	-	-	-	-	-	-	-	-	-	-
1660	(*Z*)-4-Octenylbutyrate	-	-	-	-	-	-	-	-	-	0.5	-	-
1668	Citronellyl acetate	-	-	-	-	-	-	-	-	-	0.1	-	-
1672	(*Z*)-4-Octenyl 2-methylbutyrate	-	-	-	0.1	-	-	-	-	-	-	-	-
1686	Lavandulol	-	-	-	-	-	-	-	-	-	-	-	0.1
1687	Decyl acetate	-	-	-	-	-	-	0.3	-	-	-	-	-
1687	Methyl chavicol (=Estragole)	-	-	tr	0.1	0.6	0.1	-	0.2	-	0.3	2.1	0.7
1719	Borneol	-	-	-	-	-	-	-	-	-	-	0.3	-
1726	Germacrene D	-	-	-	-	0.4	-	-	tr	-	-	0.4	-
1726	*α*-Zingiberene	-	-	-	-	-	-	-	0.4	-	-	0.2	-
1741	*β*-Bisabolene	-	-	tr	-	-	-	-	0.4	-	-	0.2	-
1755	Bicyclogermacrene	-	-	-	-	tr	-	-	-	-	-	-	-

1773	*δ*-Cadinene	-	-	-	-	-	-	-	-	-	-	-	0.1
1777	*γ*-Bisabolene	-	-	tr	-	-	-	-	0.4	-	-	-	-
1780	(*Z*)-Anethole	-	-	-	0.1	1.1	0.1	-	-	-	-	-	-
1783	β-Sesquiphellandrene	-	-	-	-	-	-	-	tr	-	-	0.1	-
1786	Kessane	-	-	tr	-	-	-	-		-	-	-	-
1786	ar-Curcumene	-	-	-	-	-	-	-	0.6	-	-	-	-
1815	Octyl hexanoate	-	-	-	1.2	-	-	3.0	0.4	0.3	1.2	0.2	-
1823	*p*-Mentha-1(7)5-dien-2-ol	-	-	-	-	-	-	-	-	-	-	-	-
1827	(E,E)-2,4-Decadienal	-	-	-	-	-	-	-	-	tr	-	-	-
1845	(*E*)-Anethole	-	-	0.1	3.1	**30.8**	3.8	0.7	1.4	0.8	0.1	0.3	0.5
1857	Geraniol	-	-	-	-	-	-	-	-	-	-	0.1	-
1864	p-Cymen-8-ol	-	-	-	-	-	0.1	-	-	2.5	-	0.1	-
1868	(*E*)-Geranyl acetate	-	-	-	-	-	-	-	-	-	0.1	0.2	-
1870	Hexanoic acid	-	-	-	tr	-	-	-	-	-	-	-	-
1900	epi-Cubebol	-	-	-	-	-	-	-	-	tr	-	-	tr
1908	Benzyl isovalerate	-	-	-	-	-	-	-	-	-	0.2	-	-
1957	Cubebol	-	-	-	-	-	-	-	-	tr	-	-	tr
1981	Phenylethyl butyrate	-	-	-	-	-	-	-	-	-	0.3	-	-
2030	Methyl eugenol	-	-	0.9	0.2	0.1	-	-	-	-	0.1	2.1	0.8
2050	(*E*)-Nerolidol	-	-	-	-	-	-	-	-	tr	-	0.1	-
2080	Cubenol	-	-	-	-	-	-	-	-	tr	-	-	-
2088	1-epi-Cubenol	-	-	-	-	-	-	-	-	tr	-	-	-
2131	Hexahydrofarnesyl acetone	-	tr	-	-	-	-	-	-	-	-	-	-
2148	Cedrol	-	-	-	-	-	-	-	-	-	tr	-	0.9
2179	1-Tetradecanol	-	-	-	-	-	-	-	-	tr	-	0.5	-

2200	trans-Methyl-isoeugenol	-	-	-	-	-	-	-	-	-	-	0.1	-
2246	Elemicine	-	-	0.7	0.1	tr	0.3	tr	0.4	0.4	0.2	0.5	1.1
2296	**Myristicine**	**-**	**10.6**	**88.0**	**15.1**	**42.9**	**83.7**	**8.9**	**24.2**	**13.8**	8.9	**62.6**	**75.2**
2384	1-Hexadecanol	-	-	-	-	-	-	-	-	-	-	0.2	-
2384	Apiole	-	-	-	-	-	-	-	-	-	0.1	0.1	0.4
2622	Phytol	-	-	-	-	-	-	-	-	-	-	0.1	-
2655	Benzyl benzoate	-	-	-	-	-	-	-	-	-	-	0.1	-
	Yield (%)	0.8	tr	0.42	3.0	0.19	0.42	4.39	0.19	0.22	1.4	0.49	1.36
	**Total**	**100**	**99.8**	**99.8**	**99.3**	**98.9**	**100**	**99.7**	**99.8**	**98.7**	**96.8**	**96.8**	**98.2**

RRI: Relative Retention Indices calculated based on the n-alkane series; **%:** Values represent Flame Ionization Detector (FID) percentages.; **tr:** Trace (< 0.1 %); **A:** *H. crenatifolium.* fruit; **B:** *H. crenatifolium* aerial parts; **C:** *H. crenatifolium* root; **D:** *H. paphlagonicum* fruit; **E:** *H. paphlagonicum* aerial parts; **F:** *H. paphlagonicum* root; **G:** *H. sphondylium* subsp. *montanum* fruit; **H:** *H. sphondylium* subsp. *montanum* aerial parts; **K:** *H. sphondylium* subsp. *montanum* root; **L:** *H. pastinacifolium* subsp. *incanum* fruit; **M:** *H. pastinacifolium* subsp. *incanum* aerial parts; **N:** *H. pastinacifolium* subsp. *incanum* root.

Table S3. Studies on the essential oils of *Heracleum* spp. fruits.

***Heracleum* species**	**PCA and HCA no**	**Main Compounds**	**Location**	**Yield (%)**	**Reference**
H. crenatifolium	1	octyl acetate (95.4%)	Konya, Türkiye	0.8	our study
H. paphlagonicum	2	octyl acetate (39.4%), myristicine (15.1%), hexyl butyrate (12.9%)	Kastamonu, Türkiye	3.0	our study
H. sphondylium subsp. *montanum*	3	octyl acetate (73.7%)	Ankara, Türkiye	4.4	our study
H. pastinacifolium subsp. *incanum*	4	hexyl butyrate (37.0%), octyl butanoate (20.6%), octyl acetate (15.6%)	Karabük, Türkiye	1.4	our study
H. pyrenaicum subsp. *pollinianum* (Bertol.) F. Pedrotti & S. Pignatti	5	octyl acetate (50.5%)	Macedonia	1.7	[41]
H. orphanidis	6	octyl acetate (84.5%)	Macedonia	2.6	[41]
H. sphondylium subsp. *ternatum* (Velen.) Brummitt	7	octyl acetate (60.2%), octyl butanoate (10.1%)	Italy	0.4	[45]
H. sphondylium subsp. *ternatum*	8	octyl acetate (54.9%), octyl butanoate (13.4%)	Italy	0.4	[45]
H. lasiopetalum	9	octyl acetate (34.5%)	Iran	0.35	[46]
H. persicum	10	hexyl butyrate (25.6%), octyl acetate (14.7%), octyl 2-methylbutanoate (12.7%)	Tehran	1.8	[37]
H. persicum	11	octyl acetate (20.5%), hexyl butyrate (17.7%), octyl 2-methylbutanoate (10.0%)	Tehran	2.1	[37]
H. persicum	12	octyl acetate (17.4%), hexyl butyrate (16.2%), octyl 2-methylbutanoate (12.0%), hexyl 2-methylbutanoate (11.9%)	Tehran	2	[37]
H. persicum	13	hexyl butyrate (27.8%), octyl acetate (22.9%)	Tehran	2.5	[37]
H. persicum	14	octyl acetate (40.8%), hexyl butyrate (26.1%)	Tehran	3	[37]

H. persicum	15	octyl acetate (28.4%), hexyl butyrate (20%)	Tehran	2.2	[37]
H. persicum	16	octyl acetate (21.1%), hexyl butyrate (17.2%)	Tehran	1.6	[37]
H. persicum	17	hexyl butyrate (19.9%), octyl acetate (18.1%)	Mazandaran	3.5	[37]
H. persicum	18	octyl acetate (22.7%), hexyl butyrate (13.8%)	Mazandaran	4.6	[37]
H. persicum	19	hexyl butyrate (43.3%), octyl acetate (12.7%)	East Azerbaijan	2.5	[37]
H. persicum	20	hexyl butyrate (33.5%)	East Azerbaijan	3.2	[37]
H. persicum	21	hexyl butyrate (23.5%), octyl acetate (17.0%)	Alborz	2.1	[37]
H. persicum	22	octyl acetate (19.0%), hexyl butyrate (15%)	Alborz	3.9	[37]
H. persicum	23	hexyl butyrate (38.5%), octyl acetate (19.0%)	Khorasan-e Razavi	3.1	[37]
H. persicum	24	hexyl butyrate (35.5%), octyl acetate (12.3%)	Gilan	4.9	[37]
H. persicum	25	octyl acetate (32.0%), hexyl butyrate (25.8%)	Yazd	2.2	[37]
H. persicum	26	octyl acetate (28.9%), hexyl butyrate (27.5%)	Golestan	4	[37]
H. persicum	27	octyl acetate (85.5%)	Samsun, Türkiye	-	[47]
H. platytaenium	28	octyl acetate (87.9%)	Manisa, Türkiye	6.5	[48]
H. siamicum Craib	29	octyl acetate (65.3%), o-cymene (10.4%)	Thailand	1.3	[49]
H. aquilegifolium CBClarke	30	*β*-pinene (22.3%), 1,8-cineole (20.3%), *β*-phellandrene (12.3%)	India	1.3	[44]
H. persicum	31	hexyl butyrate (38.9%), octyl acetate (22.3%)	-	-	[33]
H. persicum	32	hexyl butyrate (56.5%), octyl acetate (16.5%)	Iran	4	[35]
H. crenatifolium	33	octyl acetate (88.4%)	Gümüşhane-Türkiye	5.5	[50]
H. candolleanum Wight & Arn. Gamble	34	limonene (70.0%)	India	0.5	[51]
H. crenatifolium	35	octyl acetate (93.7%)	Karaman-Türkiye	3.7	[52]
H. sphondylium subsp. *ternatum*	36	octyl acetate (87.6%)	Ankara-Türkiye	5.3	[52]
H. platytaenium	37	octyl butanoate (37.7%), octyl acetate (31.6%)	Tokat-Türkiye	2	[52]

H. paphlagonicum	38	octyl acetate (31.5%), hexyl butyrate (17.0%), octyl hexanoate (10.2%)	Çankırı-Türkiye	7.4	[53]
H. sphondylium	39	octyl acetate (67.1%), octanol (16.6%)	Slovenia	1	[38]
H. sibiricum L.	40	octyl acetate (64.6%)	Serbia	1	[38]
H. sibiricum	41	octyl acetate (64.3%), octanol (21.1%)	Serbia	1.4	[38]
H. sibiricum	42	octyl acetate (73.1%)	Serbia	1.1	[38]
H. sibiricum	43	octyl acetate (57.0%)	Serbia	1.3	[38]
H. sibiricum	44	octyl acetate (62.2%), octanol (17.0%)	Serbia	0.9	[38]
H montanum	45	octyl acetate (57.5%), octanol (15.7%), octyl hexanoate (15.0%)	Slovenia	1.4	[38]
H. ternatum Velen.	46	octyl acetate (49.0%), octyl hexanoate (11.3%)	Montenegro	0.9	[38]
H. ternatum	47	octyl acetate (42.0%), octyl hexanoate (19.8%)	Montenegro	0.7	[38]
H. pyrenaicum subsp. *pollinianum*	48	octyl acetate (66.1%)	Albania and Macedonia	1.9	[38]
H. pyrenaicum subsp. *pollinianum*	49	octyl acetate (50.5%)	Albania and Macedonia	1.7	[38]
H. pyrenaicum subsp. *orsinii* (Guss,) F. Pedrotti & S. Pignatti	50	octyl acetate (36.8%), octyl hexanoate (22.1%)	Montenegro	0.6	[38]
H. pyrenaicum subsp. *orsinii*	51	octyl acetate (30.3%), octyl hexanoate (30.1%)	Montenegro	1.7	[38]
H. verticillatum Pancic	52	octyl 2-methylbutanoate (38.3%), octyl acetate (17.7%)	Serbia	1.4	[38]
H. verticillatum	53	octyl acetate (42.3%), octyl 2-methylbutanoate (22.3%)	Serbia	1.1	[38]
H. verticillatum	54	octyl acetate (47.8%), octyl 2-methylbutanoate (20.3%)	Serbia	1.6	[38]
H. orphanidis	55	octyl acetate (84.5%)	North Macedonia	2.6	[38]
H. austriacum subsp. *siifolium* (Skop.) Nyman	56	octyl hexanoate (49.8%), octyl octanoate (13.2%), octyl 2-methylbutanoate (11.0%)	Slovenia	1.5	[38]
H. crenatifolium	57	octyl acetate (93.7%)	Konya-Türkiye	3.7	[54]
H. crenatifolium	58	octyl acetate (94.9%)	Konya-Türkiye	-	[54]
H. crenatifolium	59	octyl acetate (93.6%)	Konya-Türkiye	-	[54]
H. crenatifolium	60	octyl acetate (58.9%)	Konya-Türkiye	3.4	[54]

H. platytaenium	61	octyl acetate (87.6%)	Tokat-Türkiye	5.3	[54]
H. platytaenium	62	octyl acetate (87.8%)	Tokat-Türkiye	-	[54]
H. platytaenium	63	octyl acetate (84.8%)	Tokat-Türkiye	-	[54]
H. platytaenium	64	octyl acetate (85.6%)	Tokat-Türkiye	4.9	[54]
H. sphondylium subsp. *ternatum*	65	octyl butanoate (37.7%), octyl acetate (31.6%)	Ankara-Türkiye	2	[54]
H. sphondylium subsp. *ternatum*	66	octyl acetate (38.5%), octyl butanoate (34.6%)	Ankara-Türkiye	-	[54]
H. sphondylium subsp. *ternatum*	67	octyl butanoate (40.0%), apiole (20.2%), octyl acetate (19.2%)	Ankara-Türkiye	-	[54]
H. sphondylium subsp. *ternatum*	68	octyl butanoate (41.1%), octyl acetate (25.4%), apiole (14.7%)	Ankara-Türkiye	1.3	[54]
H. sphondylium subsp. *ternatum*	69	octanol (50.3%), octyl butanoate (24.6%)	Denizli-Türkiye	3.7	[52]
H. sosnowskyi Manden.	70	methyl butanoate (14.0%), octyl acetate (13.6%), hexyl butyrate (13.3%), octanol (11.2%), o-cymene (10.3%), hexyl acetate (10.1%)	Belarus	-	[55]
H. sosnowskyi	71	octyl acetate (18.5%), hexyl acetate (13.7%)	Russia	-	[55]
H. sosnowskyi	72	Hexyl 2-methylbutanoate (11.5%)	Russia	-	[55]
H. sosnowskyi	73	octyl acetate (48.9%), methyl butanoate (11.5%)	Russia	-	[55]
H. sosnowskyi	74	octyl acetate (11.1%), hexyl 2-methylbutanoate (17.0%)	Russia	-	[55]
H. sosnowskyi	75	octyl acetate (36.7%), methyl butanoate (17.0%)	Russia	-	[55]
H. sosnowskyi	76	-	Russia	-	[55]
H. sosnowskyi	77	octyl acetate (16.2%)	Russia	-	[55]
H. lescovii Gross.	78	α-Pinene (11.5%), octyl acetate (11.4%), o-cymene (10.3%), hexyl acetate (10.1%)	Russia	-	[55]
H. asperum Mert. & WDJKoch	79	α-Pinene (45.6%)	Russia	-	[55]
H. dissectum Ledeb.	80	-	Russia	-	[55]
H. hirtum	81	octyl acetate (12.9%)	Russia	-	[55]

H. platytaenium	82	octyl butanoate (83.5%)	Türkiye	-	[56]
H. siamicum	83	octyl acetate (65.3 %), o-cymene (10.3%)	Thailand	-	[49]
H. rawianum C.C. Towns	84	hexyl butyrate (20.5%), octanol (21.7%), Isobutyl phenylacetate (13.8%)	Iran	-	[57]
H. rechingeri Manden.	85	hexyl butyrate (38.4%), octyl acetate (13.8%)	Iran	2.2	[37]
H. gorganicum	86	hexyl butyrate (33.3%), octyl acetate (18.4%), hexyl 2-methylbutanoate (10.3%)	Iran	3.1	[37]
H. persicum	87	octyl acetate (20.5%), hexyl butyrate (17.7%)	Iran	3.8	[37]
H. anisactis	88	octyl acetate (48.7%), octyl butanoate (16.8%)	Iran	0.8	[37]
H. pastinacifolium	89	octyl acetate (59.5%)	Iran	1.1	[37]
H. rawianum	90	octyl acetate (75.4%)	Iran	1.9	[37]
H. platytaenium	91	octyl hexanoate (8.8%)	Türkiye	-	[58]

Table S4. Studies on the essential oils of *Heracleum* spp. aerial parts.

Heracleum species	PCA and HCA no	Main Compounds	Location	Yield (%)	Reference
H. crenatifolium	1	octyl acetate (85.9%), myristicine (10.6%)	Konya, Türkiye	-	our study
H. paphlagonicum	2	myristicine (42.9%), (*E*)-anethole (30.8%), octyl acetate (11.3%)	Kastamonu, Türkiye	0.19	our study
H. sphondylium subsp. *montanum*	3	octyl acetate (56.1%), myristicine (24.2%)	Ankara, Türkiye	0.19	our study
H. pastinacifolium subsp. *incanum*	4	myristicine (62.6%)	Karabük, Türkiye	0.49	our study
H. persicum	5	hexyl butyrate (30.2%), octyl acetate (12.8%), ethyl decanoate (12.7%), methyl butanoate (10.6%)	Iran	-	[59]
H. moellendorffii	6	*β*-pinene (9.2%), myristicine (7.1%)	China	0.7	[26]
H. thomsonii C. B. Clarke	7	neryl acetate (36.2%), terpinolene (22.2%)	India	0.3	[60]
H. thomsonii	8	neryl acetate (51.6%)	India	-	[60]
H. pastinacifolium	9	myristicine (53.6%), trans-*α*-bergamotene (10.6%)	Iran	0.2	[33]
H. persicum	10	(*E*)-anethole (25.0%), octyl 2-methylbutanoate (14.2%), hexyl butyrate (10.0%)	Iran	0.2	[33]
H. rechingeri	11	hexyl butyrate (29.7%), octyl butanoate (10.1%)	Iran	0.2	[33]
H. rechingeri	12	elemicine (41.1%)	Iran	0.2	[33]
H. moellendorffii	13	germacrene-D (21.8%), octyl acetate (19.6%)	China	0.03	[61]
H. sibiricum	14	octyl butanoate (36.8%), hexyl butyrate (16.1%), octanol (13.6%)	Serbia	0.59	[62]
H. transcaucasicum	15	myristicine (70.0%), octanol (14.2%)	Iran	0.2	[63]
H. anisactis	16	myristicine (93.5%)	Iran	0.3	[63]
H. dissectum	17	*α*-pinene (22.1%), myrcene (10.9%)	-	0.1	[64]
H. sibiricum	18	octyl butanoate (36.8%), hexyl butyrate (16.0%), octanol (13.6%)	Serbia	0.6	[62]
H. pyrenaicum subsp. *pollinianum*	19	(*E*)-nerolidol (28.5%), germacrene-D (10.8%)	Macedonia	0.2	[41]
H. orphanidis	20	octyl acetate (83.5%)	Macedonia	0.3	[41]
H. candicans Wall. ex DC.	21	germacrene-D (29.5%), sabinene (12.4%)	India	-	[27]
H. sprengelianum (Wight and Arnott)	22	1,8-cineole (21.2%), *β*-pinene (16.2%), *β*-phellandrene (11.4%)	India	1	[44]
H. aconitifolium Woronow	23	limonene (13.9%)	-	0.1	[40]

H. voroshilovii Gorovoj.	24	*β*-elemene (10.8%), n-amyl-3-methylbutanoate (10.5%)	-	0.1	[40]
H. mantegazzianum	25	limonene (50.4%), (*E*)-anethole (20.7%)	-	0.3	[40]
H. wilhelmsii	26	limonene (60.2%)	-	0.3	[40]
H. antasiaticum	27	octyl acetate (44.7%)	-	0.2	[40]
H. leskovii Grossh.	28	limonene (15.3%), octyl acetate (12.3%)	-	0.1	[40]
H. rechingeri	29	octyl acetate (29.4%), elemicine (23.0%)	Iran	0.4	[13]
H. candolleanum	30	sabinene (13.2%)	India	0.1	[51]
H. persicum	31	(*E*)-anethole (60.2%)	Iran	1.1	[65]
H. stevenii	32	octyl acetate (30.0%), octyl octanoate (19.0%)	Russia	0.04	[42]
H. persicum	33	(*E*)-anethole (47.5%), 1-(4-methoxy phenyl)-2-propanone (18.1%), myrcene (13.5%)	Iran	0.9	[65]
H. stevenii	34	octyl acetate (35.0%), octyl octanoate (15.0%)	Russia	0.03	[42]

Table S5. Studies on the essential oils of *Heracleum* spp. roots.

Heracleum species	PCA and HCA no	Main Compounds	Location	Yield (%)	Reference
H. crenatifolium	1	myristicine (88.0%)	Konya, Türkiye	0.42	our study
H. paphlagonicum	2	myristicine (83.7%)	Kastamonu, Türkiye	0.42	our study
H. sphondylium subsp. *montanum*	3	octyl acetate (57.1%), myristicine (13.8%)	Ankara, Türkiye	0.22	our study
H. pastinacifolium subsp. *incanum*	4	myristicine (75.2%)	Karabük, Türkiye	1.36	our study
H. sprengelianum	5	1,8-cineole (23.1%), *β*-pinene (21.8%),<br>*β*-phellandrene (15.2%)	India	0.7	[44]
H. candolleanum	6	*α*-pinene (18.9%), bornylene (18.6%),<br>octyl acetate (11.9%)	India	1	[43]
H. pyrenaicum subsp. *pollinianum*	7	*β*-pinene (35.1%)	Macedonia	0.3	[41]
H. orphanidis	8	(*Z*)-falcarinol (80.0%)	Macedonia	0.1	[41]
H. transcaucasicum	9	myristicine (96.8%)	Iran	1.7	[63]
H. anisactis	10	myristicine (95.1%)	Iran	1.3	[63]
H. stevenii	11	octyl acetate (35.0%), limonene (20.0%), octyl octanoate (18.0%), octyl hexanoate (12.0%)	Russia	0.1-0.4	[42]
H. sphondylium	12	(*E*)-*β*-ocimene (28.9%)	Slovenia	0.1	[38]
H. sibiricum	13	*β*-pinene (26.2%), methyl eugenol (22.3%)	Serbia	0.4	[38]
H. sibiricum	14	methyl eugenol (21.8%), *β*-pinene (16.0%),<br>(*Z*)-falcarinol (10.6%)	Serbia	0.1	[38]
H. sibiricum	15	methyl eugenol (26.9%), *β*-pinene (21.2%)	Serbia	0.6	[38]
H. sibiricum	16	limonene (22.7%), *α*-pinene (18.7%), *β*-pinene (17.7%)	Serbia	0.9	[38]
H montanum	17	(*E*)-*β*-ocimene (20.4%)	Slovenia	0.1	[38]
H. ternatum	18	*β*-pinene (47.3%), (*E*)-*β*-ocimene (15.6%)	Montenegro	0.1	[38]
H. pyrenaicum subsp. *pollinianum*	19	*β*-pinene (39.6%), terpinolene (22.5%)	Albania and Macedonia	0.4	[38]
H. pyrenaicum subsp. *pollinianum*	20	*β*-pinene (31.5%)	Albania and Macedonia	0.3	[38]
H. pyrenaicum subsp. *pollinianum*	21	*β*-pinene (35.1%)	Albania and Macedonia	0.3	[38]
H. pyrenaicum subsp. *orsinii*	22	*β*-pinene (38.6%), (*E*)-*β*-ocimene (11.2%)	Montenegro	0.1	[38]
H. verticillatum	23	*β*-pinene (34.3%), limonene (24.8%), intermedeol (12.8%)	Serbia	0.4	[38]

H. verticillatum	24	*β*-pinene (23.5%), limonene (19.2%), intermedeol (10.9%)	Serbia	0.3	[38]
H. verticillatum	25	*β*-pinene (30.5%), limonene (16.0%)	Serbia	0.3	[38]
H. orphanidis	26	(*Z*)-falcarinol (80.0%)	North Macedonia	0.1	[38]
H. austriacum subsp. *siifolium*	27	(*Z*)-falcarinol (72.3%)	Slovenia	0.1	[38]

Table S6. *P. aeruginosa* PAO1 OD values.

Methanole Extracts	OD Control	OD MB
	**AM. (OD) ± SD**	**AM. (OD) ± SD**
H. crenatifolium (aerial parts)	1.032 ± 0.319	0.3122 ± 0.077
H. crenatifolium (roots)	1.032 ± 0.319	0.4581 ± 0.042
H. paphlagonicum (aerial parts)	1.032 ± 0.319	0.3275 ± 0.097
H. paphlagonicum (roots)	1.032 ± 0.319	0.4123 ± 0.057
H. sphondylium (aerial parts)	1.032 ± 0.319	0.9043 ± 0.416
H. sphondylium (roots)	1.032 ± 0.319	0.3520 ± 0.125
H. pastinacifolium (aerial parts)	1.032 ± 0.319	0.2766 ± 0.023
H. pastinacifolium (roots)	1.032 ± 0.319	0.3728 ± 0.053

OD: Optical Density, **OD MB:** Optical Density of Microorganism Biofilm, **AM:** *Arithmetic Mean*, **SD:** Standart Deviation

Table S7. *P. aeruginosa* PAO1 OD values.

**Hexane Extracts**	**OD Control**	**OD MB**
	**AM. (OD) ± SD**	**AM. (OD) ± SD**
H. crenatifolium (aerial parts)	0.263 ± 0.037	0.078 ± 0.001
H. paphlagonicum (aerial parts)	0.263 ± 0.037	0.068 ± 0.013
H. paphlogonicum (roots)	0.263 ± 0.037	0.244 ± 0.026
H. sphondylium (aerial parts)	0.263 ± 0.037	0.084 ± 0.003
H. sphondylium (roots)	0.263 ± 0.037	0.095 ± 0.012
H. pastinacifolium (aerial parts)	0.263 ± 0.037	0.115 ± 0.019

OD: Optical Density, **OD MB:** Optical Density of Microorganism Biofilm, **AM:** *Arithmetic Mean*, **SD:** Standart Deviation

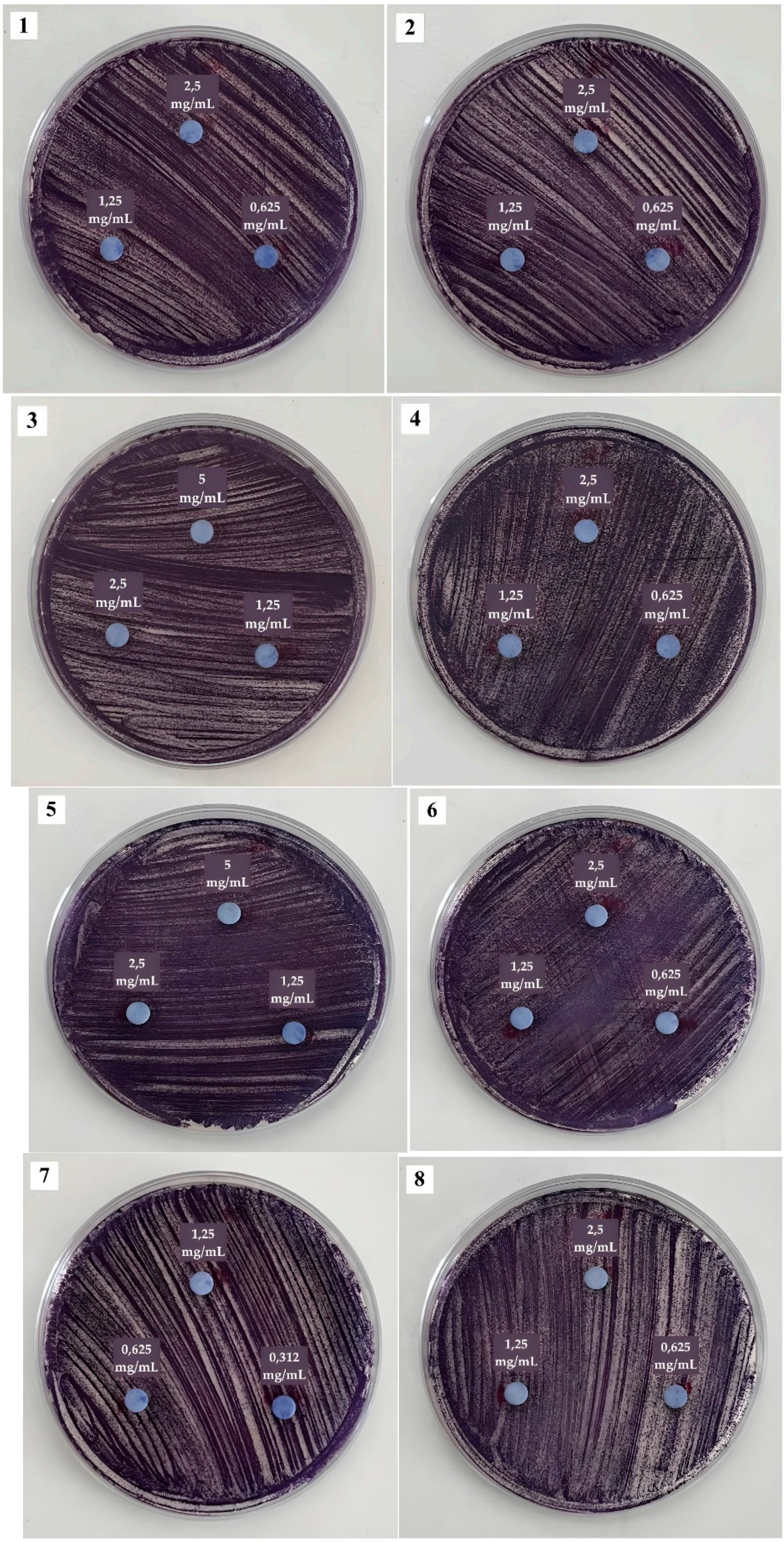

Figure S1. The anti-QS activity of methanol extracts of four *Heracleum* species (**1:** *H. crenatifolium* (aerial parts), **2:** *H. crenatifolium* (roots), **3:** *H. paphlagonicum* (aerial parts), **4:** *H. paphlagonicum* (roots), **5:** *H. sphondylium* (aerial parts), **6:** *H. sphondylium* (roots), **7:** *H. pastinacifolium* (aerial parts), **8:** *H. pastinacifolium* (roots)).

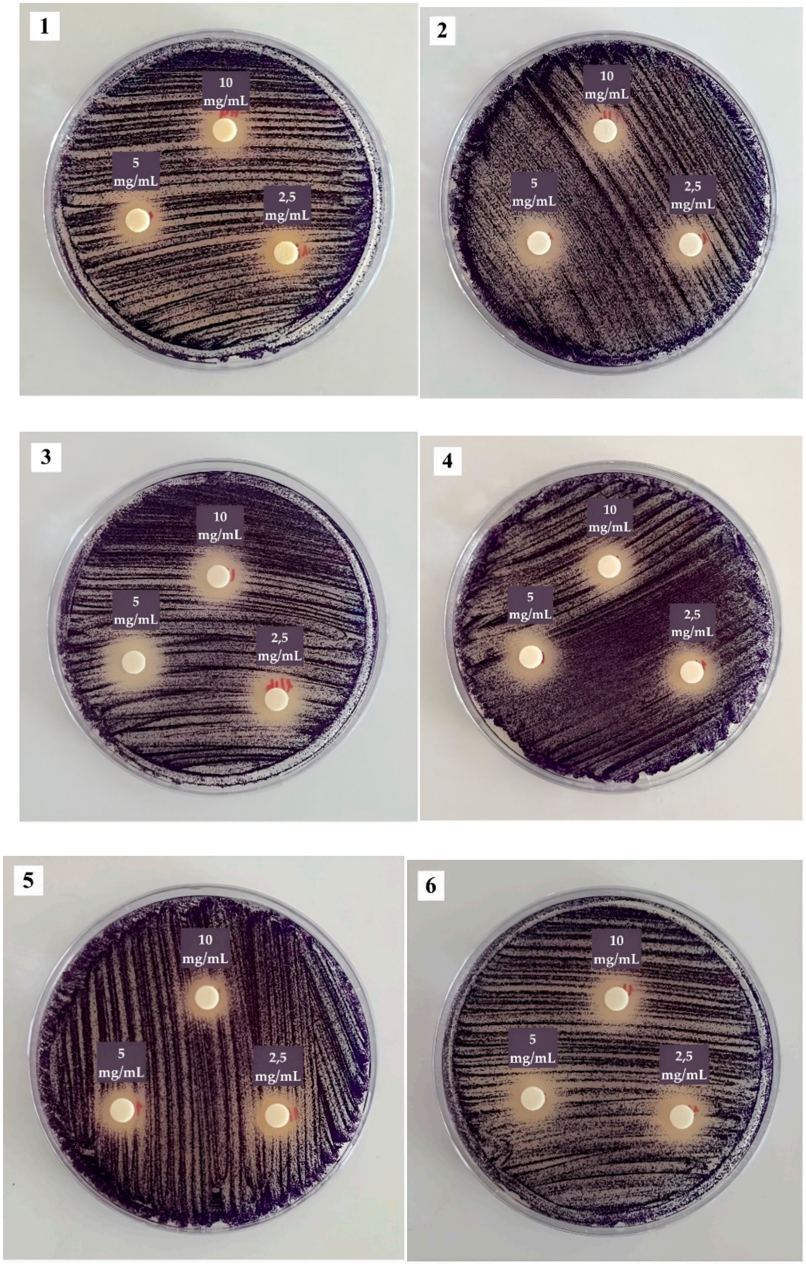

Figure S2. The anti-QS activity of *n*-hexane extracts of four *Heracleum* species (**1:** *H. crenatifolium* (aerial parts), **2:** *H. paphlagonicum* (aerial parts), **3:** *H. paphlagonicum* (roots), **4:** *H. sphondylium* (aerial parts), **5:** *H. sphondylium* (roots), **6:** *H. pastinacifolium* (aerial parts)).
